# The role of biofilm formation in the pathogenesis and antimicrobial susceptibility of *Cutibacteriumacnes*

**DOI:** 10.1016/j.bioflm.2021.100063

**Published:** 2021-12-09

**Authors:** Tom Coenye, Karl-Jan Spittaels, Yvonne Achermann

**Affiliations:** a Laboratory of Pharmaceutical Microbiology, Ghent University, Gent, Belgium; b Division of Infectious Diseases and Hospital Epidemiology, University Hospital Zurich, University of Zurich, Zurich, Switzerland

**Keywords:** *Cutibacterium acnes*, Biofilm, Antibiotics

## Abstract

*Cutibacterium acnes* (previously known as *Propionibacterium acnes*) is frequently found on lipid-rich parts of the human skin. While *C. acnes* is most known for its role in the development and progression of the skin disease acne, it is also involved in many other types of infections, often involving implanted medical devices. *C. acnes* readily forms biofilms *in vitro* and there is growing evidence that biofilm formation by this Gram-positive, facultative anaerobic micro-organism plays an important role *in vivo* and is also involved in treatment failure. In this brief review we present an overview on what is known about *C. acnes* biofilms (including their role in pathogenesis and reduced susceptibility to antibiotics), discuss model systems that can be used to study these biofilms *in vitro* and *in vivo* and give an overview of interspecies interactions occurring in polymicrobial communities containing *C. acnes*.

## 1 Introduction: from the acne bacillus over *Propionibacterium acnes* to *Cutibacterium acnes*

*Cutibacterium acnes* was first described in 1896 as the ‘acne bacillus’ found in comedones of acne patients. It was successfully cultivated in 1897 and formally named *Bacillus acnes* in 1900. Due to its morphological similarity to members of the genus *Corynebacterium*, it was reclassified as *Corynebacterium acnes* in 1923 [1]. Later studies revealed that growth of this organism is inhibited by oxygen and that it produces propionic acid as one of its main fermentation products, and this led to its transfer to the genus *Propionibacterium*, as *Propionibacterium acnes* [1]. *Propionibacterium* spp. were traditionally subdivided in ‘classic’ and ‘cutaneous propionibacteria’. The ‘classic propionibacteria’ contain species isolated from dairy products such as *Propionibacterium freudenreichii,* while the ‘cutaneous propionibacteria’ comprise *P. acnes*, *Propionibacterium avidum* and *Propionibacterium granulosum*, all isolated from human skin [2]. However, analysis of 16S rRNA gene sequences, and GC content and size of the genomes revealed marked differences between these two groups, and the genus *Cutibacterium* was proposed for the cutaneous species, resulting in the reclassification of *P. acnes* as *Cutibacterium acnes* [3].

Already in 1972 two distinct groups were recognized within this species, based on differences in cell wall composition [4]. Serological agglutination tests allowed subdivision of strains in two serotypes, referred to as type I and II. Type I strains contain glucose, mannose, and galactose as cell wall sugars, whereas galactose is not present in type II strains [4,5]. With the arrival of new techniques, new approaches were developed to group *C. acnes* isolates and understand *C. acnes* phylogeny and clonal distribution. A first method was based on the sequences of the *tly* and the *recA* genes and allowed further subdivision of serotypes I and II into phylotypes IA_1_, IA_2_, IB, IC and II, and, moreover, revealed the additional phylotype III [[6], [7], [8]]. These differences led to the proposal to reclassify the main type I, II and III phylotypes into three distinct subspecies: *C. acnes* subsp. *acnes*, *C. acnes* subsp. *defendens*, and *C. acnes* subsp*. elongatum* for phylotypes I, II and III, respectively [9,10]**.** Subsequently, two multilocus sequence typing (MLST) schemes have been described for *C. acnes*. The first is based on the partial sequencing of nine housekeeping genes and was developed in 2010 [11]. A second scheme based on seven genes was developed in 2011 and updated a year later to include eight housekeeping genes [12,13]. More recently, a single locus sequencing typing method has been developed, giving a similar discriminatory power compared to MLST while being cheaper and faster [14]. An alternative approach to grouping *C. acnes* strains is based on ribotyping. This method is based on sequence analysis of the gene coding for the 16S rRNA [15]. The resulting ribotypes (RT) are based on unique single nucleotide polymorphisms in this gene which allow to distinguish between the main phylotypes as well as between types IA_1_ and IA_2_. However, the scheme is not in full agreement with phylotyping, as for example type IB and IC share RT1 and RT5, while RT1 is also shared between type IA_1_ and IB.

## 2 Association of *C. acnes* with the human skin and its role in acne

*C. acnes* can metabolize lipids produced by the human sebaceous glands, thrives in lipid-rich environments [[16], [17], [18], [19]] and as a result, is most frequently found on the more lipid-rich parts of the human skin including the face, chest, shoulders, and scalp, reaching densities of 10^6^ colony forming units (CFU) per cm^2^ [[20], [21], [22], [23]]. In contrast, low numbers – approx. 10^2^ CFU per cm^2^ - are found on dryer areas, such as skin of the lower extremities [22]. Acne is a multifactorial disease of the pilosebaceous unit, and contributing factors include inflammation, changes in keratinization, androgen-induced increase in sebum production and *C. acnes* colonization of the follicle [24,25]. Excessive production of sebum by the sebaceous gland and hyperkeratinization of the ductal keratinocytes lead to the formation of microcomedones [26]. In these microcomedones, *C. acnes* thrives in the lipid-rich and anaerobic environment. *C. acnes* colonization and the resulting activation of the immunocompetent keratinocytes and sebocytes play essential roles in acne pathogenesis. Triglycerides present in the sebum are hydrolyzed by bacterial lipases and the resulting free fatty acids have comedogenic properties and can act as damage associated molecular patterns, while the released glycerol acts as a nutrient source for *C. acnes* [21]. Other factors produced by *C. acnes* include co-hemolytic Christie–Atkins–Munch-Peterson (CAMP) factors and porphyrins that will propagate acne pathogenesis through the generation of pro-inflammatory cytokines in human keratinocytes and sebocytes. This includes IL-1β, which is produced as a consequence of activation of the NLRP3 inflammasome [27,28]. Due to the continued sebum production and degradation of the follicular wall as a result of host degrading enzymes, pressure in the pilosebaceous unit increases, which causes the follicle to rupture, releasing all of its contents in adjacent tissue, ultimately leading to formation of superficial pustules, deeper papules or severe nodules [29]. The presence of *C. acnes* alone is not an explanation for the occurrence of acne, as the organism is present in both healthy and affected hair follicles and it seems equally unlikely that variation in relative abundance of *C. acnes* can explain the differences observed [15,24]. However, the typing methods mentioned above allow to correlate specific *C. acnes* groups with disease pathology. *C. acnes* strains belonging to phylotype IA_1_ are more often found in acneic skin, while higher phylotypic diversity as well as an enrichment of phylotype II strains can typically be found on healthy skin. In addition, recent research has demonstrated that *C. acnes* strains belonging to phylotype III dominate in progressive macular hypomelanosis [30,31]^.^ An ‘acne index’ was assigned to each RT by calculating its prevalence in acne patients. As RTs 4, 5, 8, and 10 are significantly enriched on the skin of acne patients, strains belonging to these RTs are characterized by a high acne index. In contrast, RTs 6 and 16 are strongly associated with healthy skin and therefore strains belonging to these RTs possess a low acne index [15,32]. Detailed analysis of the genome sequence of a large number of isolates belonging to these different RTs confirmed the presence of *tad* and *sag* genes (involved in adhesion and hemolysis, respectively) in RT4 and RT5, potentially providing a link between the acne index of a *C. acnes* strains and its virulence properties.

## 3 Biofilm formation by *C. acnes* and biofilm composition

Biofilm formation by *C. acnes* was first described in 1999, when it was shown that *C. acnes* forms biofilms on prosthetic hips [33]; *C. acnes* biofilm formation on various biomaterials was subsequently confirmed in a wide range of other studies (e.g. Refs. [[34], [35], [36], [37], [38], [39]]). In 2007 it was shown that several *C. acnes* strains readily form biofilms *in vitro* and that production of virulence factors like lipases is increased in biofilms compared to planktonic cells [40]. Upregulation of genes encoding virulence-associated CAMP factors [41] as well as the production of these virulence factors in a sebum-based *in vitro* biofilm model [42] were subsequently also demonstrated.

The composition of the *C. acnes* biofilm matrix has been explored in several *in vitro* studies. Biofilms of a *C. acnes* skin isolate grown in cell culture flasks contained polysaccharides, proteins and extracellular DNA (eDNA), with the polysaccharides containing α-mannopyranosyl and α-glucopyranosyl residues [41]. The matrix of biofilms of various *C. acnes* strains recovered from contaminated cardiac pacemaker devices and formed in 96-well plates was found to contain eDNA, proteins, and poly-*N*-acetyl glucosamine [43]. Finally, biofilms of an acneic *C. acnes* RT5 strain grown *in vitro* on cellulose acetate filters were found to contain polysaccharides (62.6%), proteins (9.6%), eDNA (4.0%) and other compounds (23.8%, including porphyrin precursors) [44]. In the latter study the main biofilm matrix polysaccharide was the same as that of the *C. acnes* cell wall and contained *N*-acetylgalactosamine, *N*-acetylmannosamine, 2-acetamido-2-deoxy-galactose and 2,3-di-acetamido-2,3-dideoxy-mannuronic acid residues, but no evidence for the presence of poly-*N*-acetyl glucosamine was obtained. These data suggest that overall biofilm composition is similar to what is observed in other bacterial biofilms [45] but can vary between strains, environmental conditions and/or biofilm model system. Experiments with proteinase K and DNase I revealed that both enzymes reduce attachment of a variety of *C. acnes* strains, suggesting that both eDNA and proteins are important for adhesion to abiotic surfaces, although sensitivity to DNase I was more strain-dependent [46].

## 4 Evidence for *C. acnes* biofilms *in vivo*

Jahns, Alexeyev and co-workers [20,47,48] demonstrated the presence of *C. acnes* biofilm-like structures in acne skin biopsies; such biofilms were more frequently observed in follicles of acne patients than in those of healthy controls [49]. These *in vivo C. acnes* biofilms showed different morphologies, with some attaching to the follicle wall and/or the hair shaft, while others occurred in the lumen; interestingly these different colonization patterns could be observed in the same hair follicle [48]. *C. acnes* biofilms were also observed in atherosclerotic carotid artery specimens where they are often part of a multispecies biofilm [50].

However, most evidence for a role of *C. acnes* in human disease comes from implant-associated infections [24,[51], [52], [53], [54], [55]]. The implementation of improved sampling techniques and diagnostic procedures over the last two decades has led to increased recovery of *C. acnes* from these infections and there is now convincing evidence that *C. acnes* biofilms are involved in infections related to the use of prosthetic joints, other orthopedic devices, cerebrovascular devices, breast implants, and cardiovascular devices. In all orthopedic infections, evidence of a biofilm can be found if the fluid from the sonicated implant is investigated [[56], [57], [58], [59]]. Sonication dislodges adherent bacteria off the implant while preserving microbial viability allowing to cultivate biofilm bacteria present in the sonicated fluid [60]. For example, in breast implants it was shown showed that the use of sonication allowed the detection of bacteria in 41% of removed breast implants and positive bacterial culture following sonication of the implant was correlated with the degree of capsular contracture; among the most frequently isolated organisms was *C. acnes* [37,61]. One of the criteria to confirm a periprosthetic joint infection is recovering the same pathogen in two or more intraoperative cultures, highlighting the importance of culture in diagnosis [62,63]. However, bacteria in the biofilm typically have low metabolic activities and grow slowly, and often conventional culture-based techniques fail to diagnose biofilm-related infections, unless prolonged incubation is used [64,65]. The fact that *C. acnes* from frozen stocks grows within 2–3 days in a research laboratory but requires up to 14 day to grow from orthopedic infections samples strongly suggests they are present in these samples as biofilms [66]. Among periprosthetic joint infections, *C. acnes* is the dominant pathogen found after shoulder arthroplasty [24] while *Cutibacterium avidum* dominates after hip arthroplasty [67]. Treatment includes surgical debridement and antibiotics for a prolonged time. In general, the infection free outcome after treatment of periprosthetic joint infections due to *Cutibacterium* spp. is about 85%, but worse if only a debridement is performed and the implant is retained [68]. This observation indicates that these infections are biofilm-related and that removal of the periprosthetic biofilm is needed to increase success rate. In a large multicenter study studying risk factors for *Cutibacterium* spp. relapses, radical surgery and a prolonged antibiotic treatment over 6 weeks led to the best outcomes and avoided relapse of infection [68]. This is yet another indication for the biofilm character of the infection, as a non-biofilm infection would be expected to heal with a shorter antibiotic treatment. *C. acnes* causes several cardiovascular device-related infections, such as prosthetic valve endocarditis, and pacemaker and cardiac implantable cardioverter-defibrillator infections. Infections can be divided into local infections (pocket infections) or device-related bloodstream infections, including device-related endocarditis [69]. Diagnosis can be challenging because symptoms are often subtle due to low virulence and slow growth of *C. acnes*. Endocarditis caused by *C. acnes* has been associated with both native and prosthetic valves but more often develops on valve prostheses, most commonly on the aortic valve [70]. A Swedish national registry of infective endocarditis with a search for *Cutibacterium* spp. infection between 1995 and 2016 revealed 51 episodes of prosthetic valve endocarditis of which 63% underwent surgery, suggesting a mature biofilm infection that could not be treated with antibiotics alone [71]. The presence of *C. acnes* biofilms in endocarditis has been confirmed with fluorescent *in situ* hybridization, which allows confirmation of biofilm-like structures within the histological context and rules out contamination [72]. Spondylodiscitis is an infection of the vertebral body and/or the intervertebral disc space. and is mainly caused by *Staphylococcus aureus* and *Escherichia coli* [73]; while infections with *C. acnes* are rare, they occur. In 2010, Uckay et al. reported 29 patients with spondylodiscitis caused by *C. acnes* who presented with back pain [74]. In patients with a spinal instrumentation, low-virulent microorganisms such as coagulase-negative staphylococci and *C. acnes* are typical microorganisms that are identified next to *S. aureus* or Gram-negative pathogens [75,76]. The biofilm of *C. acnes* in these cases seems to be an important virulence factor since most of the implant-associated infections requires removal of the implant to cure the infections. For example, Köder et al. found that treatment with biofilm-active antibiotics was associated with better treatment outcome and less postoperative pain intensity although this finding was not specific for *C. acnes* spine infection [77]. Also in degenerative lumbar disc disease *C. acnes* can play a role [78], and *C. acnes* biofilms have been visualized in intervertebral discs of patients undergoing microdiscectomy [79] and in samples from patients with lumbar disc herniation [80].

## 5 *C. acnes* biofilms and failure of antimicrobial therapy

Treatment with topical antibiotics (and in the past also systemic antibiotics) is often prescribed in severe cases of acne, and it is thus not a surprise that resistance in *C. acnes* has emerged worldwide [24,[81], [82], [83]]. While typically a rather sensitive organism, the long courses of antibiotics needed in acne treatment have led to colonization with erythromycin- and clindamycin-resistant strains in >50% of antibiotic-treated patients and >20% of these patients are colonized with tetracycline-resistant strains [84]. As could be expected, resistant *C. acnes* strains are currently not only found in acne patients treated with antibiotics, but also in other types of infections, including infections related to the use of various medical devices.

It is well-known that microbial biofilm formation contributes to the failure of antimicrobial therapy [85] and failure of antibiotic therapy has partly been attributed to biofilm formation by *C. acnes*. For example, substantially higher concentrations of cefamandole, ciprofloxacin, and vancomycin are required for inhibition and eradication of *in vitro* grown *C. acnes* biofilms (using polymethylmethacrylate bone cement and titanium alloys as substrates) compared to planktonic bacteria [34]. Likewise, compared to planktonic cells, *C. acnes* biofilms grown in microtiter plates were substantially less sensitive to killing by a range of anti-microbial products used for the treatment of acne, including 0.5% minocycline, 1% clindamycin, 0.5% erythromycin, 0.3% doxycycline, 0.5% oxytetracycline and 2.5–5% benzoyl peroxide [40]. Partial eradication (i.e. reduction to less than 10 CFU/ml) of *C. acnes* biofilms formed on titanium disks required prolonged exposure to penicillin (7 days), linezolid or linezolid + rifampicin (14 days), and prevention of relapse (i.e. full eradication) required a 14 day treatment of penicillin or linezolid plus rifampicin, but could not be achieved by treatment with linezolid alone [86]. Eradication of *C. acnes* from glass beads required considerably higher antibiotic concentrations than those needed for killing planktonic cells for rifampicin (4-fold higher), daptomycin (16-fold higher), vancomycin (64-fold higher) and levofloxacin (256-fold higher), while for the **β**-lactam antibiotics penicillin G and ceftriaxon the difference was smaller (2-fold higher for both) [87]. Finally, it was shown that while penicillin can easily penetrate into *in vitro* grown *C. acnes* biofilms, this is not the case for ciprofloxacin and clindamycin, and a four day treatment with the latter antibiotics at a concentration that is 50 times higher than the minimal inhibitory concentration showed no effect [41]. While the studies mentioned above suffer from the limitation that they are *in vitro* studies using surface-attached biofilms, they clearly indicate that biofilm formation has the potential to contribute to the reduced antimicrobial susceptibility observed for *C. acnes in vivo*.

## 6 Interspecies interactions in multispecies communities containing *C. acnes*

### 6.1 Interactions of *C. acnes* with other members of the skin microbiome

The human skin is home to a large number of different bacteria and the skin microbiome plays an important role in controlling colonization by pathogens and in modulating the cutaneous immune system [88,89]. While over 200 genera have been identified on the skin, more than 60% of the genera belong to the corynebacteria, cutibacteria, and *s*taphylococci. Large-scale studies on the microbiome of acneic follicles have not yet been performed, but initial data point to a dominance of *Cutibacterium* spp.*, Staphylococcus* spp. and *Malassezia* spp. (previously known as *Pityrosporum* spp.) [[90], [91], [92]].

Microorganisms colonizing the same skin area influence each other through competition for the limited amount of nutrients on the skin and through the production of antimicrobial compounds [89]. For example, short-chain fatty acids produced by *C. acnes* inhibit biofilm formation by *S. epidermidis* and *S.* *aureus* (the latter to a lesser extent), but not of *Pseudomonas aeruginosa* or *Bacillus subtilis* [93]. In addition, under specific conditions, *C. acnes* fermentation products inhibit the growth of both *S. aureus* and *S. epidermidis* [94]. Finally, the recently described thiopeptide antibiotic cutimycin, produced by *C. acnes*, reduces colonization of skin hair follicles by *Staphylococcus* species [95]. These data strongly suggest that *C. acnes*, while being an important player in the development of acne, also has a beneficial effect on the host, by limiting growth of potential pathogens on the skin.

It should however be noted that these interactions are not a one way street and antagonistic activity of *S. epidermidis* towards *C. acnes* has also been described. This could be due to the fermentation of glycerol by *S. epidermidis* leading to the production of succinic acid or other short chain fatty acids that inhibit *C. acnes* [96] and/or the secretion of other inhibitory factors, including polymorphic toxins [97]. In addition, it has been suggested that *C. acnes* biofilms may act as a ‘sanctuary’ for *S. aureus*, protecting it from harsh conditions during prolonged co-culture [98]. These interactions between *S. aureus* and *C. acnes* in polymicrobial communities are definitely not always passive, as it has been shown that *S. aureus*-induced haemolysis and cell lysis were increased when *S. aureus* was grown in the presence of *C. acnes* and that this is due to CAMP factors produced by *C. acnes* [99]. In addition, coproporhyrin III produced by *C. acnes* induces *S. aureus* aggregation and plasma-independent biofilm development on an abiotic surface; this biofilm promoting activity depends on *sarA*, a known biofilm regulator in *S. aureus* [100]. Recent work has suggested that these interactions between *C. acnes* and *S. aureus* could be co-modulated by human natriuretic peptides [101]. The latter are not the only hormones to which *C. acnes* responds, as it earlier had been shown that physiologically relevant levels of norepinephrine induce biofilm dispersion and stimulate expression of genes coding for various *C. acnes* virulence factors (including lipases and hyaluronate lyase) [50].

### 6.2 Interkingdom interactions between C. acnes and fungi

*C. acnes* can form multispecies biofilms with the dimorphic fungus *Candida albicans* [102,103]. Interestingly, unlike pathogens like *S. aureus* and *P. aeruginosa*, in these multispecies biofilms *C. acnes* adheres both to yeast cells and hyphae and the presence of *C*. *acnes* in these *in vitro* biofilms significantly reduced the susceptibility of *Candida albicans* to the antifungal agent micafungin [102]. Also this interaction seems to be beneficial to both partners, at least under specific conditions, as *Candida albicans* enhanced early *C. acnes* biofilm formation in the presence of oxygen (but not in anaerobic conditions) [103]. In the context of development of dandruff, interactions between *C. acnes* and fungi (in particular species belonging to the genus *Malassezia*) also appear to be important [104] and mixed-species biofilms of *C. acnes* and *Malassezia restricta* were observed in a pre-clinical cell-culture based dandruff model [105]. Currently the molecular basis for the interactions between *C. acnes* and fungi in these polymicrobial communities is unknown.

## 7 Model systems to study *C. acnes* biofilm formation

### 7.1 *In vitro* models

While a wide range of *in vitro* and *in vivo* biofilm models is available [106,107], most information on *C. acne*s biofilms is derived from studies in which biofilms are formed on abiotic surfaces (cell culture flask or microtiter plate) under conditions that bear little relevance for the *in vivo* situation. Recently a dynamic (flow-cell based) model was described as well [108]. Although valuable information about *C. acnes* biofilm biology can be obtained in these models, it is important to realize that such biofilms are different from *in vivo* biofilms [109]. To better mimic prosthetic joint infections, *in vitro* models in which biofilm formation on various biomaterials (including stainless steel and titanium) can be studied, have also been described [34,35,110].

**Fig. 1:**
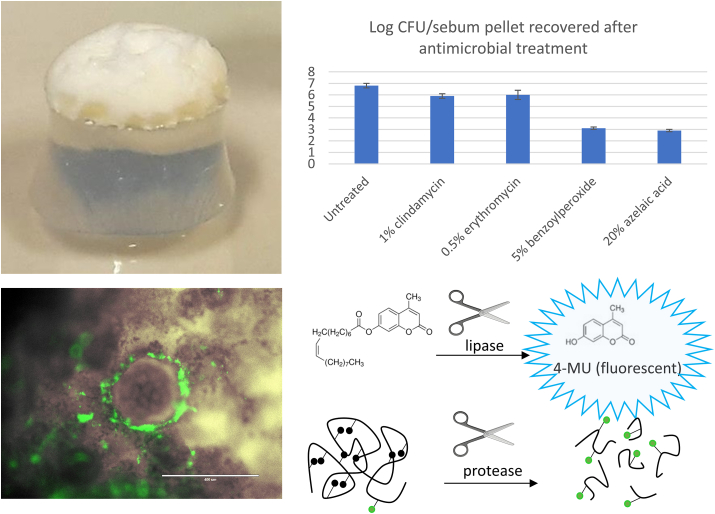
*Left*: Artificial sebum pellet on a silicone support (top) and SYTO-stained *C. acnes* biofilm model (bottom) [42]. *Right*: Examples of types of analyses that can be carried out using this model; including antimicrobial susceptibility testing (top) and quantification of production of virulence factors like lipases (middle) and proteases (bottom).

In order to allow the *in vitro* study of *C. acnes* biofilms in the context of acne in more *in vivo* like conditions, a model using artificial sebum (consisting of tripalmitin, palmitic acid, cholesterol, tocopherol acetate, triolein, jojoba oil and squalene, mixed with an equal volume of microbial growth medium) was developed. In this model *C. acnes* biofilm formation can be studied, as well as the production of virulence factors like lipases, proteases, and CAMP factors [42] (Fig. 1).

### 7.2 Cell-culture based models

Several cell-culture based models suitable to study the interaction between *C. acnes* (biofilms) and the human host are also available. As acne is an exclusively human disease, these models require the use of human cell lines [111,112].

The skin is the first line of defense against external influences and is comprised of immunocompetent cells including keratinocytes, which account for over 95% of all the cells in the epidermis [113]. The most basic form of *in vitro* keratinocytes are primary normal human epidermal keratinocytes (NHEK), directly dissociated from parental tissue and subsequently grown as monolayers. Although these cells retain the features of the original tissue, the main disadvantage of primary cells is that they will reach senescence after approx. 15–20 passages, limiting the duration and scope of experiments. In contrast, immortalized cell lines can be cultured for an extended time and their genotype and phenotype remains stable over time. These immortalized cell lines can be established after spontaneous mutations or induced by introducing viral oncogenes that affect the cell cycle [113,114]. An example of such a frequently-used cell line is the HaCaT keratinocyte cell line, a spontaneously immortalized cell line derived from a long-term primary culture of skin keratinocytes [115]. The HaCaT cell line exhibits a relative authentic phenotype, is known for its consistent growth and proliferation capacity for over 140 passages, and has been widely used (e.g. Refs. [[116], [117], [118]]). Other immortalized human epidermal keratinocytes include the NM1, NIKS, N/TERT, SV-HEK2, and SVTERT KC cell lines [[119], [120], [121], [122], [123]].

A more complex, tissue-like model is the reconstituted/reconstructed human epidermis (RHE), which requires NHEKs to obtain optimal tissue morphology i.e. a stratified epithelium. In this model, the NHEKs are cultured on a collagen matrix placed at an air-liquid interface, producing 8–12 layers of epidermis, that can be used to study infection and inflammation by various organisms, including *C. acnes* [105,114,121,124,125]. Some of these RHE models, including EpiDerm, EpiSkin and SkinEthic [[126], [127], [128]] are commercially available. RHE has recently been used to study biofilm formation of *C. acnes* (alone and in combination with *Malassezia restricta*) in a pre-clinical dandruff model [105] as well as to study the interaction between acneic skin and different phylotypes of *C. acnes* [125].

Another important cell type in the context of acne is the sebocyte, and isolation of human sebaceous glands and the culture of primary sebocytes were the first steps in the establishment of an *in vitro* sebocyte model [129]. However, due to their characteristic terminal differentiation, initiated by the accumulation of lipids until the cells burst, experiments with primary sebocytes are limited in time (3–6 passages) and prolonged experiments thus require multiple donors [112]. In order to overcome this restriction, several immortalized human sebocyte cell lines were developed of which the SZ95 cell line is the most commonly used. Originally obtained from facial sebocytes of a 87-year old woman and transfected with the simian virus-40 large T antigen, SZ95 cells retain the characteristics of normal sebocytes [130]. Other immortalized sebocyte cell lines include SEB-1 [131] and Seb-E6E7 [132].

**Fig. 2:**
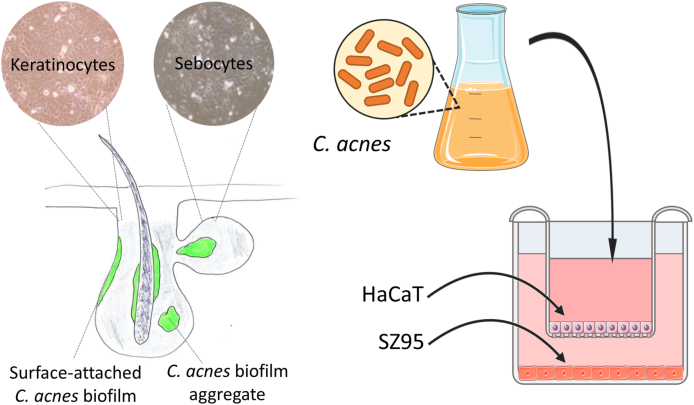
*Left*: schematic overview of the pilosebaceous unit with the localization of keratinocytes and sebocytes. *Right*: schematic overview of a *C. acnes* – keratinocyte – sebocyte co-culture model [136].

In order to include the cellular cross-talk between multiple cells of the epidermis, co-culture models have been developed in which two (or more) cell types are combined. Two main types of co-culture models exist; i.e. mixed co-cultures and segregated co-cultures [133]. These models typically result in a more *in vivo*-like morphology and more realistic environment [134]. One of the most common skin co-culture models uses keratinocytes grown on a dermal compartment containing a collagen matrix and primary normal human dermal fibroblasts [114,135]. Recently an *in vitro* co-culture model combining HaCaT keratinocytes and SZ95 sebocytes in a ‘well and insert’ system was developed [136,137]. The keratinocytes are cultivated on the membrane at the bottom of the insert, while the sebocytes are grown as monolayers in the well. After cultivation, the keratinocytes in the inserts are infected with *C. acnes* (Fig. 2). In this model, there is physical contact between keratinocytes and bacteria, whereas indirect interaction, through the production of soluble factors, is possible between the sebocytes in the well and the keratinocytes and bacteria in the insert.

**Fig. 3:**
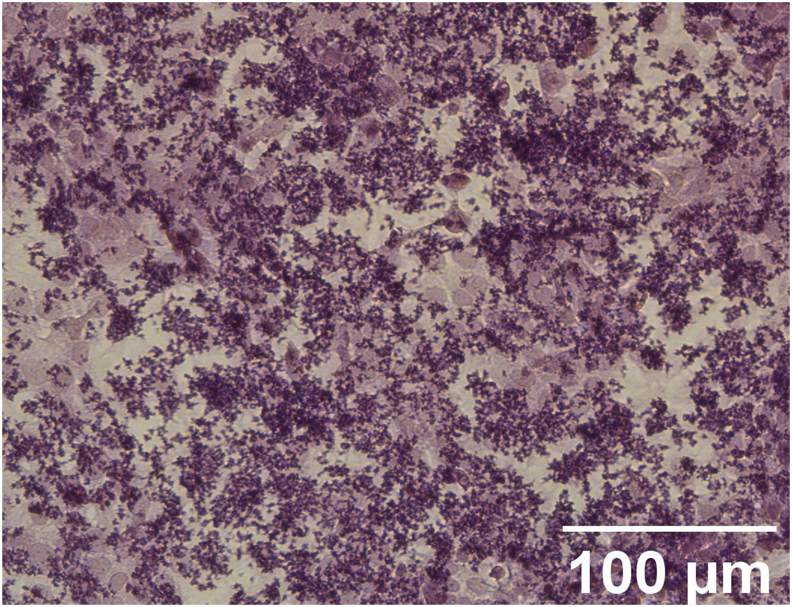
*C. acnes* biofilm aggregates associated with HaCaT cells (stained with a modified Gram stain [137]).

Several of the cell-culture based models described above were recently used to elucidate interactions between *C. acnes* and its human host. First of all, the adhesion of *C. acnes* cells and biofilm aggregates to HaCaT keratinocytes (Fig. 3) and SZ95 sebocytes, the effect of *C. acnes* on keratinocyte tight junctions in a HaCaT monoculture and in an keratinocyte-sebocyte co-culture model, and *C. acnes* invasion through the keratinocyte cell layer, were investigated, and this for a set of phylotype I and II strains [137]. A significantly higher association of (acneic) type I strains to both skin cell lines in comparison to type II strains was observed, and differences in breakdown of tight junctions (higher in type I strains) and invasion frequency (higher in type II strains) were also noted. Secondly, it was shown that acne-associated *C. acnes* strains and their porphyrin extracts activate NRLP3 inflammasome assembly leading to IL-1β release, something that is not observed in non-acneic strains [136]. These acneic strains were found to produce higher levels of porphyrins than non-acneic strains and this high porphyrin production leads to activation of the inflammasome via the induction of K^+^ leakage. These observations are in line with previous data showing that acne-associated type I clade IA-2 strains produce significantly higher levels of pro-inflammatory porphyrins than type II strains which are typically associated with healthy skin and contain the porphyrin repressor gene *deoR* [138]. However, other *C. acnes* strains that possess *deoR* (including type I clades IB-3 and IC) also produce high levels of porphyrins [139], indicating other factors must be involved as well. While the biological implications of these observed differences between different *C. acnes* (sub)groups for the pathogenesis of acne are still unclear, they reinforce the notion that there are profound and biologically-relevant differences between these (sub)groups and illustrate the power of using these cell-culture based models.

### 7.3 *In vivo* models

In order to study *C. acnes* biofilms under physiologically relevant conditions, several more advanced models have been developed. These include various animal models in which implant-associated infections can be studied, e.g. a foreign-body infection model in which polytetrafluorethylene (Teflon) cages are subcutaneously implanted in the flanks of the guinea pigs [87,140], an intramedullary nail model in rabbits [110], a model for hematogenous infection of a total knee arthroplasty in rabbits [141], a rabbit tibial implant infection model [142] and a sheep intradiscal infection model [143]. The subcutaneous cage model allowed to study the activity of various antibiotics against biofilm-associated *C. acnes* and demonstrated low cure rates for daptomycin, vancomycin, levofloxacin and rifampicin, despite good activity against planktonic cells. For eradication of these *in vivo C. acnes* biofilms, combinations of rifampicin with daptomycin (or vancomycin) were required [87]. Use of the rabbit tibial implant infection model led to the identification of 24 immunogenic *C. acnes* proteins, of which nine were exclusively produced by biofilm-grown *C. acnes* [142].

Recently a germ-free *Drosophila melanogaster* (fruit fly) model was developed to study *C. acnes*, *C. avidum* and *Cutibacterium granulosum* biofilms [144]. By maintaining the fruit flies on a lipid-rich diet, an anaerobic lipid-rich environment is created in the gut, which mimics the environment of the hair follicle. Biofilms readily form in this model, which can also be used to study therapeutic interventions (e.g. biofilm dispersal after exposure to DNase I could be demonstrated in this model).

## 8 Concluding remarks and future perspectives

*C. acnes* is a skin commensal that is also important in various infections, going from acne to device-related infections. This bacterium contains a wide range of (putative) virulence factors, and biofilm formation seems to be a common theme in many *C. acnes* infections. There is growing evidence that some *C. acnes* strains cause more damage to human cells and/or are more pro-inflammatory than others, but why that is the case is not entirely clear. While the increased production of certain virulence factors in biofilm grown *C. acnes*, as well as differences in the production of virulence factors (e.g. lipase) and pro-inflammatory mediators (e.g. porphyrins) between different *C. acnes* strains are likely to play a role, more research is needed. In addition, as a skin-associated organism, *C. acnes* frequently interacts with other organisms and how this influences biofilm formation, virulence, proinflammatory activity and cytotoxicity remains to be investigated in depth**.**

## Declaration of competing interest

The authors declare the following financial interests/personal relationships which may be considered as potential competing interests: TOM COENYE is senior editor of Biofilm. Given his role as Senior Editor, TOM COENYE had no involvement in the peer review of this article and has no access to information regarding its peer review. Full responsibility for the editorial process for this article was delegated to AKOS KOVACS.
